# Tofacitinib Adherence and Outcomes in Refractory Inflammatory Bowel Disease

**DOI:** 10.1093/crocol/otab075

**Published:** 2021-10-23

**Authors:** C Alex Wiles, Nisha B Shah, Jake Bell, Baldeep S Pabla, Elizabeth A Scoville, Robin L Dalal, Dawn B Beaulieu, David A Schwartz, Sara N Horst

**Affiliations:** 1 Department of Internal Medicine, Vanderbilt University Medical Center, Nashville, Tennessee, USA; 2 Vanderbilt University Medical Center, Vanderbilt Specialty Pharmacy, Nashville, Tennessee, USA; 3 Department of Gastroenterology, Hepatology, & Nutrition, Vanderbilt University Medical Center, Nashville, Tennessee, USA

**Keywords:** tofacitinib, ulcerative colitis, Crohn’s disease, adherence

## Abstract

**Background:**

Tofacitinib has been approved for moderate-to-severe ulcerative colitis and studied in Crohn’s disease. Understanding medication adherence to oral medications in severe disease is essential.

**Methods:**

We retrospectively reviewed adherence and real-world outcomes of inflammatory bowel disease patients who initiated tofacitinib at a single care center. Adherence was measured by proportion of days covered.

**Results:**

Sixty-three patients were identified. All patients failed at least one prior biologic therapy. Mean proportion of days covered was 95.7% for ulcerative colitis and 93.1% for Crohn’s disease. Significant clinical and endoscopic response was seen.

**Conclusion:**

Adherence was high in a cohort with highly refractory disease.

## Graphical Abstract

**Figure:**
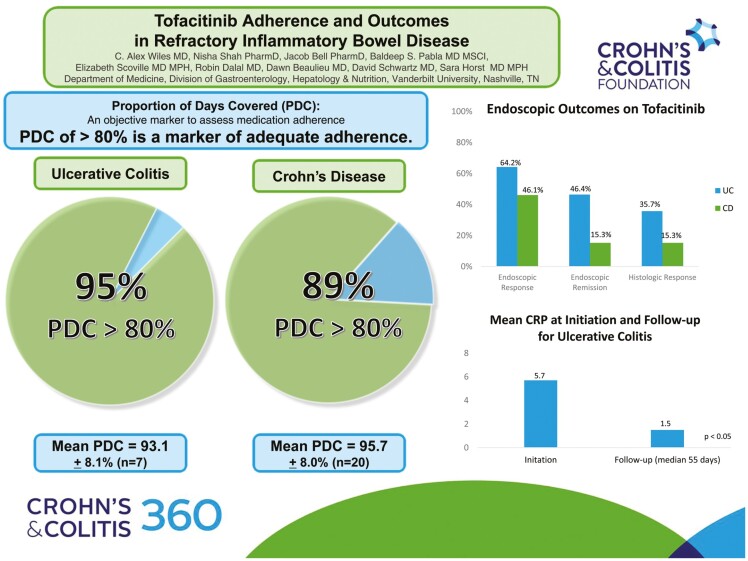

## Introduction

Inflammatory bowel diseases (IBDs), including Crohn’s disease (CD) and ulcerative colitis (UC), are chronic conditions characterized by relapsing inflammation of the gastrointestinal tract. Tofacitinib is an oral, small-molecule Janus kinase (JAK) inhibitor. JAKs are intracellular tyrosine kinases that activate signal transducers and activators of transcription (STAT), thereby inducing gene transcription and production of pro-inflammatory cytokines. Given the potential of JAK–STAT inhibition to dampen the dysregulated immune response observed in IBD, it has been studied for use in patients with CD or UC.^1^

Tofacitinib has shown benefit over placebo in patients with moderate-to-severe UC. Three phase III trials (OCTAVE 1, 2, and Sustain) demonstrated significantly improved rates of remission after 8 weeks of induction and sustained remission at 52 weeks.^1^ Further studies have demonstrated evidence that tofacitinib is both safe and effective for moderate-to-severe UC.^2^

While the efficacy of tofacitinib in patients with UC is well-established, significant improvement in disease remission was not seen in phased trials in CD. However, tofacitinib has demonstrated potential efficacy via improvement in clinical biomarkers, such as C-reactive protein (CRP) and fecal calprotectin.^3^ In smaller studies, tofacitinib has also been used in the management of patients with refractory CD.^2, 3^

Tofacitinib is the first oral medication approved for the management of moderate-to-severe UC and continues to be studied in off-label use in CD. Tofacitinib offers benefit to patients given the ease of administration of oral medication. However, as with other oral medications, there is a need to understand real-world medication adherence. Proportion of days covered (PDC) is a validated and often preferred method of measuring medication adherence.^4, 5^ PDC is defined as the total number of days of medication dispensed during an observation period divided by the number of days a medication was prescribed during the observation period. The threshold for optimal medication adherence for most chronic medications is 80%.^4, 5^

Limited data exist on adherence to tofacitinib therapy when used for the treatment of IBD. The primary aim of this study was to evaluate adherence to tofacitinib in patients with severe and refractory IBD receiving care within an integrated care center. The study also evaluated clinical, endoscopic, and histologic outcomes of patients with IBD.

## Materials and Methods

This was a retrospective cohort analysis of all patients with IBD who initiated tofacitinib within our tertiary care outpatient clinic from May 2018 to May 2020. Clinical information was obtained by chart review of IBD clinic visits, hospitalization within our health system, and also outside hospital records.

The primary outcome, medication adherence, was measured using PDC for patients who received tofacitinib through the institution’s integrated specialty pharmacy. PDC was calculated as the number of days covered by medication fills divided by the total number of days during the observation period.

Outcomes related to medication use included adverse events, medication persistence (duration of therapy in days), medication discontinuation, and reason for discontinuation. Clinical outcomes included CRP (within 30 days of initiation and in follow-up), new prednisone use, IBD-related hospitalizations, and surgeries, as well as endoscopic response and remission rates. Endoscopic response and remission rates were determined on assessment by the performing gastroenterologist, in which response or remission between endoscopies was detailed in each report (if ≥60 days from tofacitinib initiation). Endoscopic data are included for all those patients who underwent endoscopy while on tofacitinib for more than 60 days during the study period. Histologic remission (inactive chronic inflammation or normal-appearing tissue) as determined by pathologist review was also obtained.

For patients with UC, patient-reported outcomes (PROs) were collected at baseline and longitudinally at each visit, specifically Simple Clinical Colitis Activity Index (SCCAI) for disease activity and Short IBD Questionnaire (SIBDQ) for health-related quality of life.

Statistical analysis was performed using Wilcoxon signed-rank matched-pairs test for nonparametric measures.

### Ethical considerations

Institutional review board approval at Vanderbilt University Medical Center was obtained for this retrospective chart review.

## Results

Of 62 patients included, 43 patients had UC, 84% with pancolitis, and 19 patients had CD. Patient demographics are detailed in Table 1. For patients with UC, all patients had failed at least one biologic agent and 84% (36/43) of patients had failed 2+ previous biologic therapies, indicating refractory disease. Median follow-up was 293 days (range 37–2038 days). Mean PDC was 96 ± 8% in the 20 patients with available data with only one patient having PDC less than 80%. Four patients experienced adverse events, including leukopenia, liver test abnormalities, arthralgias, or infection. Seventeen patients discontinued tofacitinib, most commonly due to lack of or loss of response (33%, 14/43) and the rest for adverse events (7%, 3/43). During the follow-up period, 8 patients were hospitalized, and 14 patients required prednisone. Seven patients eventually underwent surgery (colectomy), and all were in the group that was hospitalized prior to colectomy. No patients were treated with another biologic agent while taking tofacitinib. Of the 28 patients who underwent endoscopic evaluation during the study period at a median of 151 days (79–272 days), the endoscopic response rate was 64% (18/28), with endoscopic and histologic remission rates of 46% (13/28) and 36% (10/28), respectively. Over a median follow-up time of 55 days (range 31–196), a significant decrease in CRP was observed (from median 5.7 [0.3–71] to 1.5 [0.1–38.4], *n* = 22), *P* < .05. A significant improvement was observed in SCCAI scores (median 7 [0–20] to 4 [0–9], *n* = 28), *P* < .05 and SIBDQ scores (median 47 [18–66] to 56 [21–63], *n* = 28), *P* < .05 (Table 2).

**Table 1.** Demographics for patients with ulcerative colitis or Crohn’s disease.

Total patients, *n*	Ulcerative colitis (*n* = 43)	Crohn’s disease (*n* = 19)
Age (years, range)	36 (21–70)	36 (19–72)
Follow-up duration (days, range)	293 (37–2038)	347 (60–1053)
Time on tofacitinib (days, range)	203 (35–2038)	248 (45–917)
Female (*n*, %)	25 (58%)	8 (42%)
BMI (kg m^–2^, range)	25 (16–67)	26 (17–51)
Prior biologic history (*n*, %)	43 (100%)	19 (100%)
Multiple biologic history (*n*, %)	36 (84%)	19 (100%)
Crohn’s disease features (*n*, %)		
Stricturing		9 (47%)
Fistulizing		10 (53%)
Perianal disease		5 (26%)
Prior IBD-related surgery (*n*, %)		9 (47%)

Abbreviations: BMI, body mass index; IBD, inflammatory bowel disease.

**Table 2.** Biomarkers and patient-reported outcome scores at tofacitinib initiation and follow-up for patients with ulcerative colitis.

	Baseline	Follow-up	Days follow-up	*P*
Clinical biomarkers (total patients = 22)				
CRP	5.7 (0.3–71)	1.5 (0.1–38.4)	55 (31–196)	<.05
ESR	20.5 (1–102)	14.5 (1–206)	55 (31–196)	NS
Patient-reported outcomes (total patients = 28)				
SCCAI	7 (0–20)	4 (0–9)	47 (30–196)	<.05
SIBDQ	47 (18–66)	56 (21–63)	47 (30–196)	<.05

Abbreviations: CRP, C-reactive protein; ESR, Erythrocyte Sedimentation Rate; SCCAI, Simple Clinical Colitis Activity Index; SIBDQ, Short IBD Questionnaire.

Of the 19 patients with CD, 58% had large bowel disease only. 42% were female and 42% with prior IBD surgery. Of 19, 17 (89%) had failed 3 prior biologic therapies, indicating that the majority of patients had refractory disease. Median follow-up was 347 days (range 60–1053). Mean PDC was 93 ± 8% in 7 patients with available data with only one patient with PDC less than 80%. Two patients experienced adverse events, with one patient developing infection and one with severe headaches. Overall, 8 patients discontinued tofacitinib (42%), most commonly due to lack of response (32%, 6/19). During the follow-up period, 7 patients were hospitalized, and 3 patients required prednisone. No patients underwent surgery. No patients were treated with another biologic agent while taking tofacitinib. Of the 13 patients who underwent endoscopic evaluation during the study period at a median of 112 days (75–262) showed 6 patients had endoscopic response (6/13, 46%) and 2 patients had endoscopic and histologic remission (2/13, 15%). Endoscopic outcomes are detailed in Table 3.

**Table 3.** Endoscopic and histologic outcomes with tofacitinib.

Total patients, *n*	Ulcerative colitis (*n* = 28)	Crohn’s disease (*n* = 13)
Median time to endoscopy (days, range)	151 (79–272)	112 (75–262)
Endoscopic response (*n*, %)	18/28 (64.2%)	6/13 (46.1%)
Endoscopic remission (*n*, %)	13/28 (46.4%)	2/13 (15.3%)
Histologic response (*n*, %)	10/28 (35.7%)	2/13 (15.3%)

## Discussion

Tofacitinib therapy offers many benefits to patients with moderate-to-severe IBD. Currently, all other therapies for moderate-to-severe disease require self-injection or infusion at an outpatient clinic; as an oral therapy, tofacitinib offers improved ease and convenience of administration. However, the adherence rate to tofacitinib in patients with IBD has not been significantly evaluated.

Encouragingly, our data demonstrate high adherence to tofacitinib in patients with moderate-to-severe IBD receiving care within an integrated care center. Mean PDC was 95% and 93% for patients with UC and CD, respectively, both values exceeding the established threshold of 80%. Only one patient in each cohort had a PDC of less than 80%, resulting in adherence rates of 95% for UC (19/20) and 86% for CD (6/7). This is important in a group of patients with severe disease, as nonadherence to medications in IBD has shown to increase the risk of disease recurrence and flare.

There is little data currently in the literature regarding adherence to tofacitinib in patients with IBD; therefore, it is useful to review prior data regarding currently available oral medication. Prior to tofacitinib, the mainstay oral medications approved for maintenance of remission for UC were 5-Aminosalicylates (5-ASA) and only for use in mild to moderate disease. An analysis of claims data from over 5000 patients on 5-ASA found PDC to be 48 ± 31% with only 21% of patients having PDC more than 80%.^6^ Other studies report wide ranges of adherence (taking >80% of doses) from 21% to 97%. A systematic review in 2010 found that most of the 17 studies reviewed reported adherence to all oral medications to be 55%–70%.^7^ A single-center study by Kane et al^8^ found that the median amount of medication consumed by UC patients on 5-ASA was 71% (8%–100%). Even with 5-ASA medications, patients with more severe disease were more likely to be adherent.^6^ Azathioprine is an oral immunomodulator used in the treatment of moderate-to-severe CD and UC (though its use in IBD is not FDA-approved) and therefore is typically used in cases of more severe disease than 5-ASA. Objective data regarding adherence to azathioprine in IBD are sparse. Some studies have assessed adherence objectively with Medication Possession Ratio (MPR) that functions similarly to PDC. Recent studies have reported adherence (MPR >80%) to azathioprine in patients with IBD between 67% and 77%.^9, 10^ The patients in our study had higher adherence than prior reports for oral medications in IBD; however, all had moderate-to-severe and refractory disease with multiple prior medication failures. Therefore, it is possible that patients with more severe disease will have a shift toward higher adherence to try to improve current symptoms or avoid recurrence of severe disease.

Adherence specifically to tofacitinib has been studied previously in patients with rheumatoid arthritis (RA), with a lower mean PDC reported 0.53–0.68.^11, 12^ However, a recent study comparing patients with IBD and RA found that IBD patients demonstrated significantly higher mean PDC and proportion of patients with PDC more than 80% with infliximab, suggesting adherence to the same medications differs between disease states.^11^ Again, this may be a difference in the threshold of acceptance of symptoms, as all patients with UC on tofacitinib would have experienced moderate-to-severe UC symptoms and failure to prior medications prior to starting medication. Indeed, our patient cohort had refractory IBD, with all patients having failed one or more biologic agents prior to tofacitinib initiation and therefore may have higher motivation to be adherent.

Patients in our cohort with UC and refractory disease still demonstrated excellent rates of endoscopic response (64%), endoscopic remission (46%), and histologic remission (36%). This is similar to findings in OCTAVE Sustain (outcomes at 52 weeks for patients who responded to tofacitinib) which found mucosal healing (Mayo endoscopic subscore *<*1) rates of 34.3%–40.6% depending on the dose.^1^ Two retrospective studies found endoscopic remission rates of 38% (5/13 patients with UC only) and 54% (7/13 patients with either UC or CD), respectively, which is similar to our cohort.^13, 14^ Patients in our study with UC had statistically significant improvement in both CRP and PROs, consistent with prior studies.^1, 15^

We were also able to show a small subset of patients with severe refractory CD failing conventional biologic and immunomodulatory therapies were able to maintain on tofacitinib and had endoscopic response. Of the 19 patients with CD, 17 had failed 3 prior biologic therapies. Despite the refractory nature of their disease, tofacitinib was safe and 58% were able to maintain on tofacitinib throughout the follow-up period. Furthermore, endoscopic response was reported in 46% of patients, though rates of endoscopic and histologic remission (each 15%) were less robust.

This study had a retrospective study design, small sample size, inability to evaluate certain details such as patient vs provider preference for discontinuation of medication, and was limited to a group of patients with refractory disease seen at an IBD-specialty single center, potentially limiting generalizability. Adherence was measured using PDC based on prescription fill data. While there is potential limitations to this, it is an objective measure of adherence and has been shown to be more accurate than other measures such as patient-reported adherence.^16^ It is unlikely that a patient would continue to refill and accumulate a medication systematically over a period of months and continue to avoid administration. Adherence is only reported for those patients who filled at our specialty pharmacy on-site; however, prior work by our center showed that those filling at other specialty pharmacies had similar demographic data.^10^

## Conclusion

Our study found high adherence to tofacitinib therapy in patients with refractory IBD receiving care within an integrated care center. Further multicenter studies are needed to evaluate the impact of high adherence on clinical outcomes in this population.

## Data Availability

Data are not publicly available but can be provided upon request.
